# Lateral-Only Flexible Intramedullary Nailing for Pediatric Tibial Shaft Fractures: An Early Comparative Experience With a Novel Technique to Reduce Symptomatic Implant Prominence

**DOI:** 10.1016/j.jposna.2026.100440

**Published:** 2026-09-01

**Authors:** Alan D. Villegas Meza, Matthew Allen, Brendon C. Mitchell, Mohammad Diab, Jaclyn Hill

**Affiliations:** 1 University of California San Francisco School of Medicine, San Francisco, CA, USA; 2 The Steadman Philippon Research Institute, Vail, CO, USA; 3 Department of Orthopaedic Surgery at the University of California San Francisco, San Francisco, CA, USA

**Keywords:** Pediatric tibial shaft fracture, Flexible intramedullary nailing, Implant prominence, Lateral-only technique, Hardware irritation, Pediatric orthopaedic trauma

## Abstract

**Background:**

Standard medial-lateral (ML) flexible intramedullary nailing (FMN) for pediatric tibial shaft fractures is associated with high rates of symptomatic hardware prominence along the subcutaneous medial border. A lateral-only (LO) technique may reduce these symptoms by avoiding medial subcutaneous hardware.

**Methods:**

We conducted a retrospective two-cohort comparison (2015-2024) at two tertiary children’s hospitals within a single health system, evaluating 42 patients (aged 5-16 years) treated with FMN for tibial shaft fractures: 13 who underwent LO fixation and 29 who underwent ML fixation. One additional patient treated with a single lateral-entry nail was excluded from the comparative analysis and is described separately; a sensitivity analysis including this patient yielded unchanged results. The primary outcome was symptomatic implant prominence, defined as implant-related complaints or clinician-documented soft-tissue findings. Secondary outcomes included operative time for the index procedure, implant removal at the last follow-up, and the incidence of complications other than implant prominence (malunion, nonunion requiring surgery, refracture, delayed union, wound complications, or growth disturbance). Given the modest sample, secondary outcomes were regarded as exploratory. Group comparisons were performed using Fisher’s exact tests and t-tests.

**Results:**

LO fixation was associated with markedly less symptomatic implant prominence than ML fixation (8% vs 66%; *P* < .001). No secondary outcome reached statistical significance. Implant removal at the last follow-up occurred in 31% of LO cases versus 55% of ML cases (*P* = .19). Among cases with a recorded surgical case time, mean operative time was 26 min shorter with LO fixation (97 vs 123 min; 95% confidence interval: −2 to 53; *P* = .063). Complications other than implant prominence were more frequent in the LO cohort (3 of 13 [23%] vs 1 of 29 [3%]) but did not differ significantly (*P* = .08) and lacked a consistent technique-specific mechanism.

**Conclusions:**

In this early comparative experience, LO FMN was associated with markedly less symptomatic implant prominence than conventional ML fixation. The secondary findings of a lower removal rate and shorter operative time are hypothesis-generating and require cautious interpretation given confounding by surgeon, era, and follow-up duration. The technique appears viable in appropriately selected patients, but prospective, adequately powered studies incorporating patient-reported outcomes are needed before comparative superiority can be established.

**Key Concepts:**

(1) Lateral-only (LO) flexible intramedullary nailing significantly reduces symptomatic implant prominence compared with conventional medial-lateral fixation for pediatric tibial shaft fractures.
(2) The lateral approach leverages anterior compartment muscle coverage at the proximal entry site to eliminate medial subcutaneous hardware and reduce implant-related morbidity.
(3) No secondary outcome reached statistical significance. The removal, operative-time, and complication comparisons are exploratory and confounded by surgeon, era, and follow-up duration.
(4) Complications other than implant prominence were numerically more frequent in the LO group, so any reduction in symptomatic prominence must be weighed against that trade-off.
(5) Flexible nailing by either entry strategy should be applied cautiously in heavier or near-mature adolescents, in whom rigid implants may be more appropriate.

**Level of Evidence:**

III

## Introduction

Tibial shaft fractures are among the most common long-bone injuries in children, accounting for approximately 15-21% of long-bone fractures [[1], [2], [3], [4]]. In younger children, these injuries frequently result from low-energy mechanisms such as playground falls and recreational sporting activities. Higher-energy trauma predominates in older children and adolescents, including motor vehicle collisions [[1], [2], [3], [4]]. Stable and well-aligned fractures can be managed with closed reduction and casting [5]. Surgical stabilization is indicated for fractures refractory to cast management or otherwise significantly displaced, unstable, open, or polytraumatic [[6], [7], [8], [9], [10], [11], [12], [13]].

In skeletally immature patients, flexible intramedullary nailing (FMN) is the standard of care for operative management of pediatric tibial shaft fractures because it avoids violation of the proximal tibial physis, minimizing the risk of physeal injury and growth disturbance. Compared with plate fixation, FMN offers smaller incisions, less soft-tissue dissection, and minimal disruption of the fracture environment while reliably achieving high union rates [6,8,14,15].

The conventional medial-lateral (ML) technique uses nails inserted from both the medial and lateral cortices to achieve divergent three-point fixation within the medullary canal. However, this approach is commonly associated with implant prominence and overlying soft-tissue irritation at the medial insertion site [[16], [17], [18]]. Implant removal rates for the traditional approach exceed 65%, with a mean time to removal of approximately six months [16,19]. It is important to note, however, that removal of flexible nails is frequently planned or elective, and reported removal rates therefore reflect a composite of surgeon preference, institutional custom, and symptomatic prominence rather than symptoms alone. Symptomatic prominence may nonetheless necessitate earlier and unplanned reoperation [9,16,20,21]. A lateral-only (LO) technique, which inserts both nails through the lateral cortex with divergent precontouring to achieve three-point fixation without medial cortical purchase, has demonstrated favorable outcomes in pediatric forearm and humeral shaft fractures [19,[22], [23], [24], [25], [26], [27], [28], [29], [30], [31]]. In the tibia specifically, this approach is anatomically advantageous: nail ends at the lateral entry site are covered by the anterior compartment musculature, providing a natural soft-tissue buffer that is absent at the subcutaneous medial border. The lateral approach also avoids the saphenous nerve, which courses along the medial tibial border and is at risk with medial entry-site dissection [27,[32], [33], [34],[35], [36], [37], [38], [39]]. The most directly analogous prior evidence comes from all-lateral versus ML retrograde nailing of pediatric femur fractures, where the all-lateral technique has been reported to shorten operative time with equivalent fracture alignment and complication rates [28]. This femoral precedent has not, however, been evaluated in the tibia. To date, no systematic studies have evaluated LO FMN for pediatric tibial shaft fractures [19,[22], [23], [24], [25], [26], [27]].

We therefore describe our early experience with LO FMN and test the hypothesis that, by eliminating medial subcutaneous hardware, it reduces symptomatic implant prominence compared with conventional ML fixation in pediatric tibial shaft fractures. We also provide exploratory comparative data on operative efficiency, implant removal, and complication profile.

## Methods

### Study design and setting

We conducted a retrospective cohort study of consecutive pediatric patients treated with FMN for tibial shaft fractures between December 2015 and May 2024 at two tertiary children’s hospitals within a single academic health system. This study was approved by the institutional review board of the participating institution, with a waiver of informed consent granted due to its retrospective design.

### Participants

Cases were identified by querying institutional electronic medical records using Current Procedural Terminology (CPT) codes associated with tibial medullary nailing procedures (27759, 27756, 27752, and 27750). Patients were excluded if they had pathological fractures, congenital deformities, a diagnosed syndrome, or incomplete clinical or radiographic documentation. The full selection process is summarized in Fig. 1. Candidate patients for the LO technique were those with diaphyseal tibial shaft fractures requiring FMN in which the canal geometry permitted two divergent laterally introduced nails; comminuted, markedly length-unstable, and very proximal or distal metaphyseal patterns were treated at surgeon discretion.

**Figure 1:**
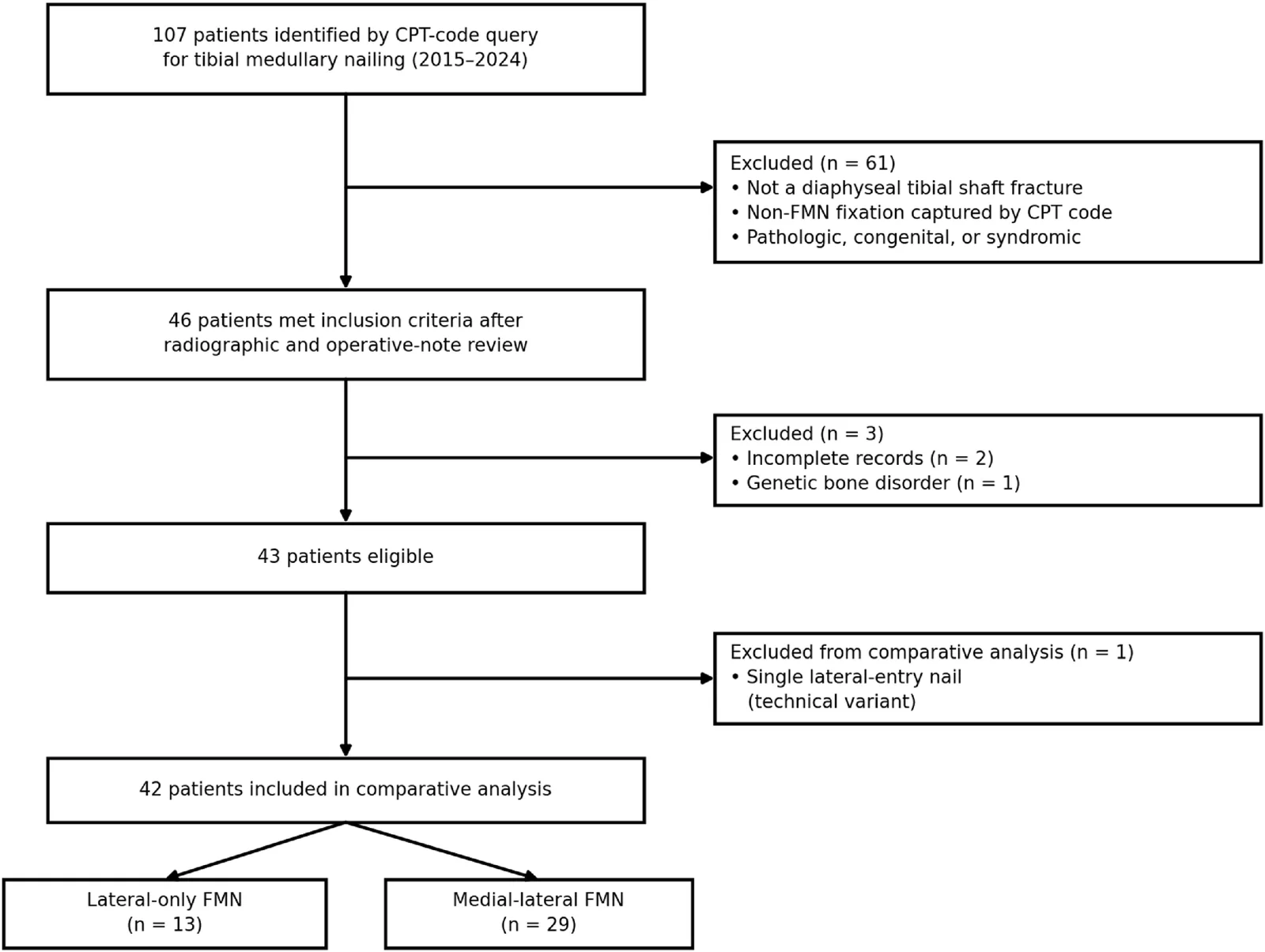
Patient selection and enrollment flow diagram. Of 107 patients identified by CPT-code query, 46 met inclusion criteria after radiographic and operative-note review and 43 were eligible after 3 further exclusions; the single-lateral-nail case was excluded from the comparative analysis, yielding 42 patients (13 lateral-only, 29 medial-lateral). *CPT, Current Procedural Terminology; FMN, flexible intramedullary nailing*.

### Intervention classification and prominence definitions

Postoperative radiographs were independently reviewed to classify fixation technique as either standard ML or LO FMN. LO fixation consisted of two drill holes for two entry sites. One additional patient received a single 4.0-mm lateral-entry nail, chosen over two 2.0-mm nails for rigidity given the patient’s weight and size after preoperative canal measurement. This single-nail case was not retained in the comparative analysis; it is noted here as a technical variant, and a sensitivity analysis including it is reported below. All conclusions are unchanged by its inclusion.

Symptomatic prominence was categorized as subjective or objective. Subjective prominence was defined as implant-related complaints (eg, pain, irritation) spontaneously reported by the patient or parent and documented in the clinical record. Because this was a retrospective study, no prospective, standardized prominence questionnaire was administered; ascertainment relied on the routine, structured postoperative examination documented at each institution rather than a study-specific checklist. Objective prominence was defined as physical examination findings exceeding expected implant contours, specifically documented by the clinician as skin tenting, bursa formation, or threatened soft-tissue integrity. For patients treated with the ML technique, the location of prominence (medial vs lateral) was recorded. Prominence assessment was based on documentation at routine postoperative visits. Radiographic union was defined as the presence of bridging callus on at least 3 of 4 cortices on orthogonal views. Delayed union was defined as failure to achieve this radiographic threshold by 3 months, and nonunion as the persistence of a visible fracture line or lack of progressive callus formation at 6 months postoperatively.

### Outcomes

The primary outcome was symptomatic implant prominence, encompassing both subjective and objective prominence, as defined earlier. This outcome was selected a priori to directly test the anatomical rationale of the LO technique. Secondary outcomes included operative time for the index fixation procedure (defined as the recorded surgical case time; cases without a recorded surgical case time were excluded from operative-time analyses), implant removal at the last follow-up, and major structural and bone-healing complications. Removal is reported at the last follow-up rather than at a fixed one-year landmark because routine elective removal of flexible nails commonly occurs between 6 and 12 months, making a fixed landmark arbitrary. Major structural complications were defined as malunion (>5° coronal angulation and/or >15° procurvatum and/or any recurvatum), nonunion requiring osteosynthesis, or refracture. Bone-healing complications were separately defined and included delayed union and oligotrophic nonunion, as described earlier. Implant-related complications were assessed independently from bone-healing events. For the between-group comparison, major structural complications, bone-healing complications, unplanned reoperation for a wound complication, and growth disturbance were combined into a composite of complications other than implant prominence. All patients underwent routine postoperative follow-up until fracture healing. At approximately 3-6 months postoperatively, documented discussions regarding elective implant removal were conducted. Patients without symptoms or planned removal were subsequently discharged from routine orthopaedic follow-up.

### Surgical technique

All procedures were performed within a single academic health system by 10 fellowship-trained pediatric orthopaedic surgeons with intraoperative fluoroscopic guidance. The proximal tibial physis was identified fluoroscopically and marked on the skin. A linear anterolateral incision was made distal to the physis. The crural fascia was incised approximately 1 cm lateral to the tibial crest, and the anterior compartment musculature was elevated subperiosteally to expose the proximal tibial cortex.

A proximal tibial entry portal was created using an oblique drill trajectory. Two flexible intramedullary nails were selected and precontoured prior to insertion. In most cases, the nails were bent into a smooth “C” configuration; when additional fracture control or spread was desired, one nail was contoured with an “S” bend to optimize three-point fixation and improve fracture stability.

Nail diameter was selected under fluoroscopy at the tibial isthmus with a target of approximately 80% combined canal fill, divided between two nails (ie, ∼40% of the canal diameter per nail). Standard techniques were then used for nail advancement, fracture reduction, and final positioning. Distally, the nail tips were advanced to rest just proximal to the distal tibial physis (metaphyseal region), avoiding physeal violation.

Proximally, the nails were cut to leave approximately 1-2 cm of nail external to the cortex to facilitate later removal. Because the anterior compartment musculature overlies the proximal nail ends, a slightly longer cut than is typically used can be tolerated with reduced symptomatic prominence. The anterior compartment muscles were allowed to fall back over the nail ends, and the crural fascia was repaired. A controlled ankle motion boot was used postoperatively for comfort. A representative comparison of the ML and LO constructs is shown in Fig. 2.

**Figure 2:**
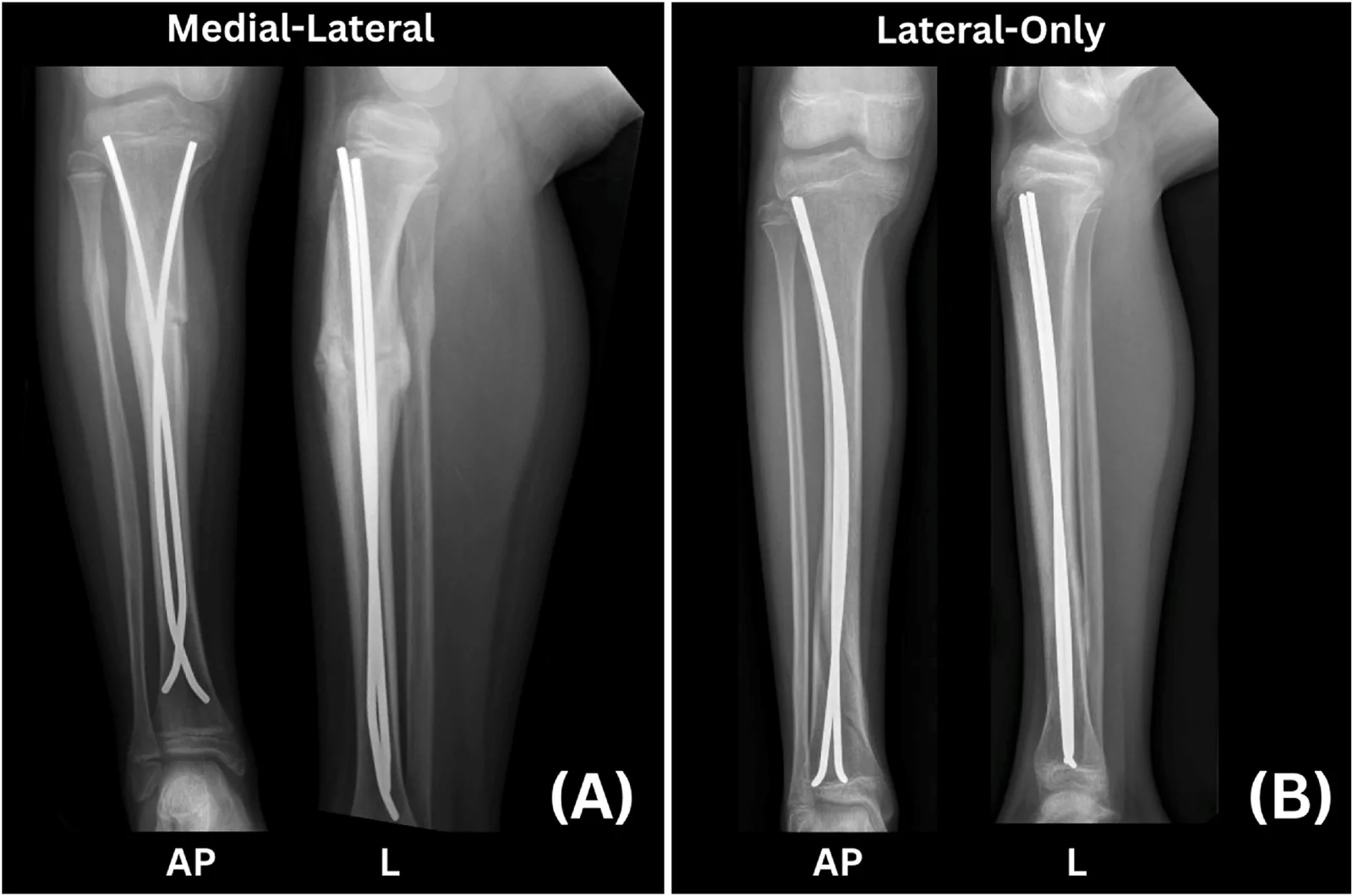
Representative anteroposterior (AP) and lateral (L) radiographs of (A) a 10-year-old male with right proximal tibial and fibular fractures treated with medial-lateral flexible intramedullary nailing, 16 weeks postoperatively, and (B) a 10-year-old female with a right distal tibial fracture treated with lateral-only flexible intramedullary nailing, 8 weeks postoperatively.

### Statistical analysis

Categorical variables were compared using two-sided Fisher’s exact tests. Continuous variables were analyzed using Student’s t-tests or Wilcoxon rank-sum tests, depending on normality.

Associations between implant prominence and reoperation were quantified using absolute risk differences (ARDs), number needed to remove (defined as the reciprocal of the ARD in implant removal, ie, the number of patients with a given prominence finding required to account for one additional removal), and odds ratios (ORs), each with corresponding 95% confidence intervals (CIs). The ARD was defined as ARD = p_1_ − p_2_, where p represents the proportion of patients undergoing implant removal. CIs for ARDs were computed using the Wald method with pooled standard error. All statistical analyses were performed using R version 4.4.0 ($ Foundational for Statistical Computing, Vienna, Austria) and Python version 3.14.7 (Python Software Foundati, Beaverton, OR, USA), with SciPy v1.17. Power calculations were performed using the statsmodels package v0.14 in Python.

### Power analysis

A prospective sample size calculation was performed prior to data collection. Published rates of implant-related prominence and reoperation for the ML approach exceed 65% [16,19]. To detect a 35-percentage-point absolute reduction (from 65% to 30%) with two-sided α = 0.05 and 80% power, the required sample sizes were as follows: equal allocation (1:1), 31 patients per group (62 total); unequal allocation (1:2), 15 LO and 30 ML patients (45 total). A 5% attrition buffer increased the final targets to 33 per group for equal allocation (66 total) and 16 of 32 for unequal allocation (48 total). Calculations used the NormalIndPower module in statsmodels, with an effect size of h ≈ 0.72. The final comparative cohort (*n* = 42; 13 LO and 29 ML patients) fell short of the 45-patient base requirement for a 1:2 allocation (88% of the attrition-buffered target of 48). Because the observed primary endpoint effect greatly exceeded the design assumption, the primary comparison remained decisive; the study is underpowered for the secondary outcomes and complication comparisons.

### Sensitivity analysis

The comparative analysis comprises the 13 double-lateral patients. To assess the influence of the excluded single-lateral-nail case, a sensitivity analysis was conducted including that case (14 LO patients; ML group unchanged at 29). Results were unchanged in direction and significance: symptomatic prominence 1 of 14 (7%) vs 19 of 29 (66%; Fisher’s exact *P* < .001), and implant removal 29% vs 55% (*P* = .12). Statistical conclusions were identical with or without the single-nail case.

## Results

### Enrollment

CPT-code query identified 107 potentially eligible patients treated between 2015 and 2024. Following radiographic and operative-note review, 46 patients met the inclusion criteria. Three were subsequently excluded due to incomplete records (*n* = 2) or a diagnosis of underlying genetic bone disorder (*n* = 1), leaving 43 eligible patients. One patient treated with a single lateral-entry nail was excluded from the comparative analysis and is described separately as a technical variant, yielding a final comparative cohort of 42 patients: 13 treated with the LO technique and 29 with ML fixation (Fig. 1). The 61 cases excluded during chart review did not meet inclusion criteria, most commonly because the CPT query captured nondiaphyseal fractures or non-FMN procedures or because the fracture was pathologic, congenital, or syndromic. Baseline characteristics, including age, skeletal maturity, sex, fracture laterality, fracture type, fracture pattern distribution, and fracture location, were similar between groups (Table 1). The only significant baseline difference was mean follow-up duration, which was shorter in the LO group (254 ± 140 days) than in the ML group (429 ± 322 days; mean difference: 175 days, 95% CI: 35-315; *P* = .017).

**Table 1:** Baseline characteristics of the study cohort.

Characteristic	Medial-lateral (*n* = 29)	Lateral-only (*n* = 13)	*P* value
**Age (y)**	11.4 ± 2.8	12.6 ± 2.9	.23
Sex—female	5 (17%)	4 (31%)	.42
Sex—male	24 (83%)	9 (69%)	
**Laterality**—**left**	11 (38%)	9 (69%)	.096
Laterality—right	18 (62%)	4 (31%)	
**Fracture type**—**oblique**	17 (59%)	7 (54%)	.90
Fracture type—spiral	5 (17%)	3 (23%)	
Fracture type—transverse	7 (24%)	3 (23%)	
**Fracture location**—**distal third**	11 (38%)	8 (62%)	.45
Fracture location—middle third	17 (59%)	5 (38%)	
Fracture location—proximal third	1 (3%)	0 (0%)	
**Fibula fracture present**	20 (69%)	9 (69%)	1.00
**Skeletally mature**	6 (21%)	5 (38%)	.27
**Mean follow-up (d)**	429 ± 322	254 ± 140	.017

*SD, standard deviation*.

*Values are shown as mean ± SD for continuous variables and as number (percentage) for categorical variables*. *Pvalues arecalculatedfrom Welch t-tests for continuous variables and Fisher’sexact tests for categorical variables (Fisher-Freeman-Halton for three-category variables). No baseline characteristic differed significantly between groups except mean follow-up duration*.

Eleven patients (26%) were skeletally mature (6 of 29 ML and 5 of 13 LO patients). Although FMN is most often used in skeletally immature patients, these adolescents were treated with flexible nails on the basis of canal diameter permitting adequate fill, fracture pattern amenable to load-sharing fixation, and surgeon judgment that the physeal-sparing benefit and lower soft-tissue burden outweighed the greater rigidity of a locked adult nail. This selection is discussed further in the text as a determinant of the study’s complication profile.

### Implant prominence and reoperation

Subjective implant prominence was reported in 1 of 13 LO cases (8%) compared with 19 of 29 ML cases (66%; Fisher’s exact *P* < .001). Objective prominence was documented in 0 of 13 LO cases and 19 of 29 ML cases (66%). Among patients undergoing implant removal, documented prominence was more common in the ML cohort (13 of 16 removals vs 1 of 4 removals; Appendix Table A1).

Across the comparative cohort, implant removal occurred in 70% (14/20) of patients with subjective prominence, compared with 27% (6/22) of those without, corresponding to an ARD of 43% (95% CI: 15-70%) and an OR of 6.2 (95% CI: 1.6-23.8; *P* = .012). Objective prominence was associated with a numerically higher rate of implant removal (63% vs 35%), corresponding to an ARD of 28% (95% CI: −1 to 58%) and an OR of 3.2 (95% CI: 0.9-11.4; *P* = .12). When both subjective and objective prominence were present, implant removal occurred in 67% of patients compared with 33% of patients with one or neither finding, corresponding to an ARD of 33% (95% CI: 5-62%) and an OR of 4.0 (95% CI: 1.1-14.6; *P* = .06). Point estimates were similar across the prominence categories, with widely overlapping CIs; the full associations are reported in Appendix Table A1.

### Operative time

Among cases with a recorded surgical case time (19 of 29 ML and all 13 LO cases), mean operative time was shorter in the LO group (97 vs 123 min), a mean difference of 26 min that did not reach statistical significance (95% CI: −2 to 53; *P* = .063; Fig. 3). Excluding open fractures, the difference was similar in magnitude and also not significant (97 vs 120 min; *P* = .12; Appendix Table A2). This comparison should be interpreted with caution: surgical case time was unavailable for 10 ML cases, and operative time is confounded by surgeon and era, which we could not disentangle from entry-point choice. We therefore make no claim of a technique-specific efficiency benefit.

**Figure 3:**
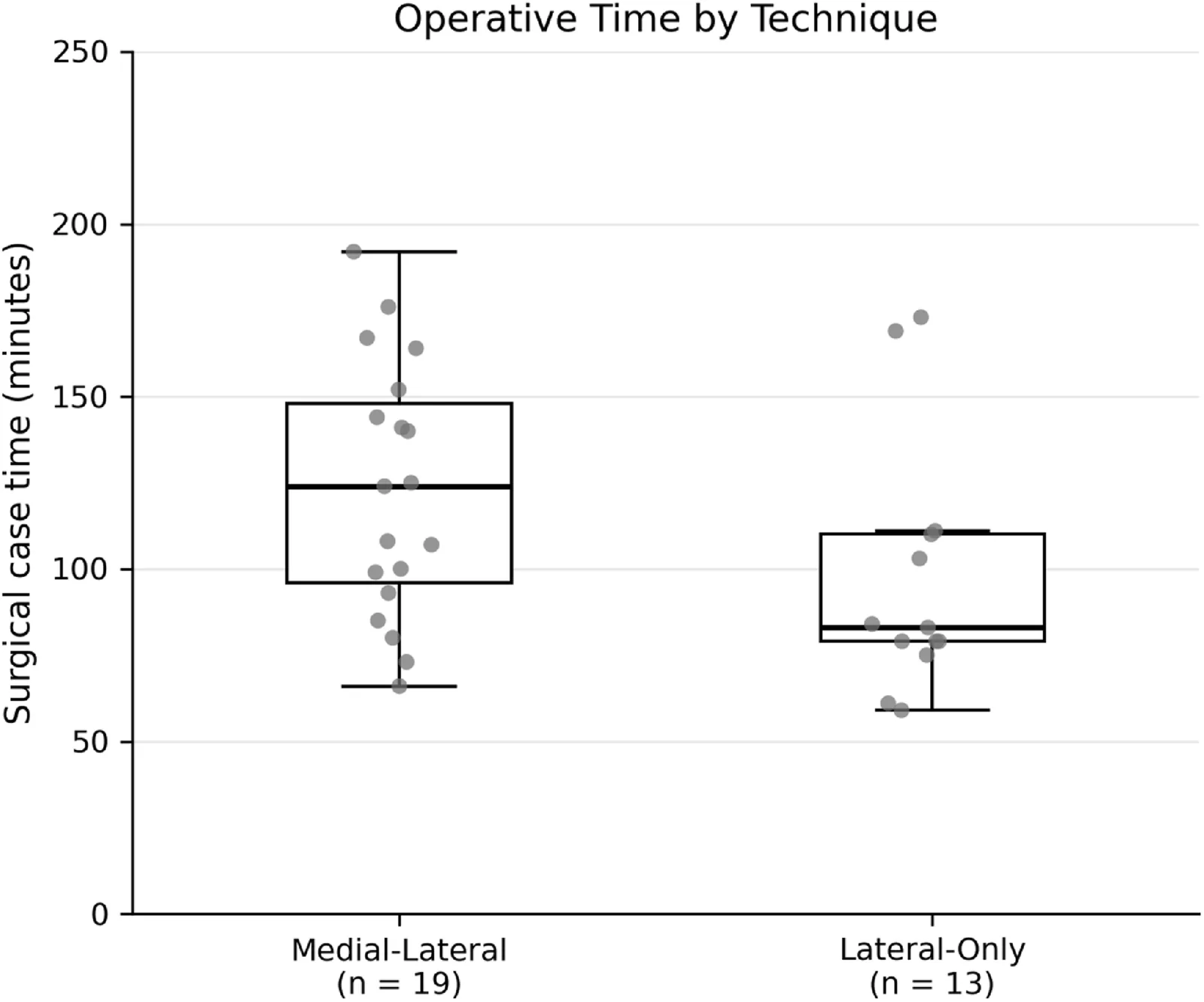
Box-plot comparison of recorded surgical case times by technique (medial-lateral *n* = 19 and lateral-only *n* = 13 cases). Boxes show the median and interquartile range (IQR); whiskers extend to 1.5× IQR; points are individual cases. The 26-min mean difference did not reach statistical significance (Welch *P* = .063).

### Implant removal

Implant removal at the last follow-up had occurred in 4 of 13 patients (31%) treated with LO fixation compared with 16 of 29 (55%) treated with ML fixation, an absolute reduction of 24 percentage points that did not reach statistical significance (95% CI: −7 to 55%; OR: 0.36, 95% CI: 0.09-1.44; Fisher’s exact *P* = .19), consistent with the study being powered for the primary prominence endpoint rather than for removal. Of the 4 LO removals, 1 (at 350 days) was performed for implant-related symptoms, 1 (at 292 days) was performed electively for family preference after fracture healing, and 2 occurred as part of complication management (wound dehiscence at 53 days; revision of an oligotrophic nonunion at 124 days). In the ML group, 13 of 16 removals (81%) were associated with documented implant prominence. Because removal reflects a composite of symptoms, surgeon recommendation, and family preference, and because our institutions did not follow a single protocol for recommending removal, we were unable to reliably separate how often removal was recommended from how often it was performed; this distinction should be captured prospectively. Among patients undergoing removal, the mean time to removal was earlier in the LO group (205 ± 139 vs 346 ± 118 days; *P* = .13).

### Complications

Implant-related symptoms were the most frequent adverse event. Subjective implant prominence or irritation was documented in 20 of 42 patients (48%), predominantly in the ML group (19 events), compared with only one event in the LO group (8%).

Complications other than implant prominence were more common in the LO cohort, though overall infrequent, and did not differ significantly (3 of 13 [23%] vs 1 of 29 [3%]; Fisher’s exact *P* = .08). Three complications occurred in the LO cohort: one wound dehiscence requiring irrigation and debridement, one oligotrophic nonunion requiring revision to rigid intramedullary nailing, and one delayed union treated with bone stimulation, with fracture healing confirmed within one month. In the ML group, one non-implant-related complication was observed: a limb-length discrepancy of less than 1.5 cm.

All 29 ML patients achieved radiographic union. In the LO cohort, 12 of 13 patients (92%) achieved union without structural complication. One patient developed an oligotrophic nonunion requiring conversion to rigid intramedullary nailing; this patient subsequently achieved osseous stability, but the postoperative course was complicated by a deep infection following the rigid-nailing salvage procedure, resulting in a prolonged time to final union. Because this infection followed the revision procedure rather than the index LO FMN, it is reported as part of this nonunion cascade rather than as a separate index-procedure complication; if it were instead counted as a fourth major event in the LO group, the cohort complication rate would be 4 of 13 (31%) and the between-group difference would reach nominal significance (*P* = .03). No deep infection occurred as a direct complication of an index FMN in either group. All patients in both cohorts ultimately achieved union.

## Discussion

This comparative analysis represents, to our knowledge, the first clinical description of an LO FMN technique for pediatric tibial shaft fractures. The primary endpoint, symptomatic implant prominence, was markedly reduced in the LO group (8% vs 66%; *P* < .001), a finding consistent with the anatomical rationale for bypassing the medial subcutaneous soft-tissue envelope. No secondary outcome reached statistical significance; the numerically lower removal rate and shorter operative time are encouraging but should be regarded as hypothesis-generating rather than confirmatory. The retrospective design, nonrandomized treatment assignment, and modest sample size limit causal inference.

The operative-time comparison warrants particular caution. There is no compelling technical reason that entry-point choice alone should materially shorten a flexible-nailing procedure, and the observed difference is most likely confounded. Although the LO technique was used throughout the study period (the earliest cases in this series were LO), 9 of 13 LO procedures occurred in the final four study years, and the technique was concentrated among a subset of surgeons: five of the 10 surgeons performed LO fixation, and a single surgeon accounted for six of the 13 LO cases while performing none of the ML cases. Operative time is therefore entangled with individual surgeon practice and with secular changes in perioperative workflow over the decade of study, and surgical case time was unavailable for 10 of 29 ML cases. We were unable to adjust for surgeon or era given the sample size, and we therefore make no claim of a technique-specific efficiency benefit. Even if a modest efficiency difference exists, published operating room cost estimates of approximately $36-$37 per minute would make it economically relevant in high-volume pediatric centers; these estimates are derived from general hospital cost analyses and may not reflect pediatric-specific institutional costs [[40], [41], [42]].

Our results should be interpreted against the published experience with conventional ML tibial flexible nailing. In the largest meta-analysis of pediatric tibial FMN (28 studies, 835 patients), the pooled major complication rate was 6.3%, with malunion in 8.5%, delayed union in 3.8%, nonunion in 1.4%, deep infection in 1.0%, and symptomatic hardware in 5.1% [20]. Our complication events fall broadly within these ranges, and all fractures ultimately united. Notably, our 66% rate of symptomatic prominence in the ML group greatly exceeds the 5.1% pooled rate of “symptomatic hardware,” a discrepancy that reflects definitional differences: we captured any patient- or parent-reported implant complaint at the subcutaneous medial border, a more sensitive threshold than the discrete complication flag used in most retrospective series and one that aligns more closely with the >65% implant-removal rates reported for this technique [16,19]. Removal per se is a limited outcome, whereas malunion, nonunion, and infection are the more clinically meaningful events. However, the present study is not powered to compare those rates.

From an anatomical perspective, the LO approach avoids the medial tibial cortex and associated subcutaneous soft tissues, which are commonly implicated in postoperative implant irritation [[16], [17], [18]]. By eliminating medial hardware prominence, the LO technique offers a plausible mechanistic explanation for the reduced symptom burden observed in this cohort. Biomechanically, the technique departs from the classic mediolateral construct in which two opposed entry points create symmetric, balanced three-point fixation across the canal. An LO construct instead relies on maximizing divergence of two precontoured nails introduced from a single cortex so that three-point contact is achieved through nail curvature and apposition against the opposite endosteal cortex rather than through opposed cortical entry. This is mechanically less symmetric and, in principle, may provide less rotational and coronal-plane control, which is a legitimate concern and may contribute to the higher number of structural complications observed in the LO group. While this strategy appears to preserve stability in appropriately selected fractures, careful patient selection remains important, particularly in older or heavier adolescents [19,[22], [23], [24], [25], [26], [27],[43], [44], [45], [46], [47], [48], [49]].

A theoretical concern with any unilateral lateral-entry technique is hardware crowding at the lateral cortex, which could trade medial prominence for lateral prominence. The present data do not support this concern: objective lateral prominence was documented in 0 of 13 LO patients, and only 1 of 13 (8%) reported any subjective symptoms. The anatomical basis for this observation lies in the muscle coverage at the proximal lateral entry site, where the anterior compartment musculature overlies the nail ends and provides a natural soft-tissue buffer that the medial subcutaneous border lacks.

The complication profile is the central caution of this study. Implant-related symptoms were substantially more common in the ML cohort, whereas complications other than implant prominence were more frequent in the LO cohort (3 of 13 vs 1 of 29. respectively). Although this difference did not reach statistical significance (*P* = .08), the point estimate is not reassuring, and with the single postrevision infection counted as a fourth event, the difference would be nominally significant (*P* = .03). We therefore cannot exclude that any reduction in symptomatic prominence is partly offset by a higher rate of more serious complications, and this trade-off must be weighed explicitly when considering the technique. The events were heterogeneous and lacked a consistent technique-specific mechanism. The single nonunion occurred in an older adolescent near skeletal maturity with a larger body habitus, a patient profile that likely exceeds the appropriate biomechanical envelope for flexible fixation; this observation, together with the 26% of the cohort who were skeletally mature, underscores that flexible nailing by either entry strategy should be applied cautiously in heavier or near-mature adolescents, in whom rigid implants may be more appropriate. This case is best understood as a patient-selection issue rather than a technique failure.

The significantly shorter mean follow-up in the LO cohort (254 vs 429 days; *P* = .017) is relevant to interpretation in two ways. First, part of the difference is attributable to a subset of ML patients who returned for subsequent unrelated orthopaedic procedures, thereby extending their documented follow-up beyond the fracture-care episode; because implant removal itself generates additional visits, the higher removal rate in the ML group also mechanically lengthened that group’s recorded follow-up, so follow-up duration and removal rate are not independent. Second, shorter follow-up in the LO group could underascertain late complications and late removals. All patients completed routine postoperative follow-up through fracture healing and the standard implant-removal discussion at 3-6 months; nonetheless, longer prospective follow-up is needed to confirm the removal findings.

Several limitations should be acknowledged. The retrospective design limits causal inference and introduces potential for selection and documentation bias. Surgical technique was not randomized; technique selection reflected individual surgeon preference, and unmeasured differences in patient selection, surgeon, or fracture characteristics cannot be fully excluded. Symptomatic prominence was assessed from clinical documentation rather than a standardized prospective instrument, which may have led to underascertainment of asymptomatic cases; however, this limitation would be expected to attenuate rather than inflate the observed group difference. The study did not include patient-reported outcome measures (PROMs). Body habitus could not be reliably abstracted for the full cohort, so body mass index could not be assessed as a potential confounder of implant prominence or complication risk. Enrollment fell just short of the prespecified 1:2 allocation target, leaving the secondary outcomes and complication analyses underpowered. Implant removal decisions reflect a composite of clinical symptoms, surgeon recommendations, and patient preferences, limiting their interpretability as a standalone endpoint. The study was not powered to detect differences in nonunion incidence. Future prospective studies incorporating PROMs, standardized prominence grading, a protocol-driven implant removal algorithm, and stratification by fracture complexity and skeletal maturity, ideally through a multicenter design or an expanded institutional cohort, would substantially strengthen causal inference.

## Conclusion

LO FMN was associated with a marked and statistically significant reduction in symptomatic implant prominence compared with conventional ML fixation for pediatric tibial shaft fractures, consistent with the anatomical rationale for avoiding the medial subcutaneous soft-tissue envelope. This early comparative experience supports the prominence finding as robust, but no secondary outcome reached statistical significance, and the numerically lower removal rate and shorter operative time may be confounded by surgeon, era, and follow-up duration. The apparent reduction in symptomatic prominence must also be weighed against a numerically higher rate of complications other than implant prominence in the LO group. Despite this, the LO technique appears to be a viable option in appropriately selected patients, particularly those at higher risk for medial hardware-related symptoms and with canal geometry and skeletal maturity suited to flexible fixation. Prospective, adequately powered, multicenter studies incorporating PROMs and standardized prominence assessment are needed to confirm these findings, characterize the complication trade-off, and refine patient-selection criteria before comparative superiority can be claimed.

## Ethics approval and consent

The authors declare that patient consent was not required; all radiographic images included in the article are fully deidentified, and no identifying information is included.

The authors declare that no patient consent was necessary as no identifying information is included in the article.

## Funding

The authors of this article declare that they did not receive funding.

## Author contributions

**Alan D. Villegas Meza:** Writing – review & editing, Writing – original draft, Visualization, Methodology, Formal analysis. **Matthew Allen:** Writing – review & editing, Writing – original draft, Formal analysis, Data curation. **Brendon C. Mitchell:** Writing – review & editing, Supervision, Conceptualization. **Mohammad Diab:** Writing – review & editing, Supervision, Conceptualization. **Jaclyn Hill:** Writing – review & editing, Supervision, Project administration, Data curation, Conceptualization.

## Declaration of competing interests

The authors declare the following financial interests/personal relationships which may be considered as potential competing interests: Jaclyn Hill reports a relationship with Stryker Orthopaedics and BridgeBio that includes consulting or advisory. If there are other authors, they declare that they have no known competing financial interests or personal relationships that could have appeared to influence the work reported in this paper.
