# Supplementary material for: Lateral-Only Flexible Intramedullary Nailing for Pediatric Tibial Shaft Fractures: An Early Comparative Experience With a Novel Technique to Reduce Symptomatic Implant Prominence
Source: J Pediatr Soc North Am. 2026 Sep 1;17:100440. doi: 10.1016/j.jposna.2026.100440 (PMC13717033; doi:10.1016/j.jposna.2026.100440)
Supplement: Supplementary file 1 [file mmc1.docx]

**Supplementary Material**

The following tables are provided as supplementary material.

**Supplemental Table S1. Association between nail prominence and implant removal**

| **Prominence Category** | **Implant Removal (Yes/Total)** | **Removal Rate % (95% CI)** | **ARD** | **NNR** | **Odds Ratio (95% CI)** |
| --- | --- | --- | --- | --- | --- |
| Subjective | 14 / 20 | 70 (46–88) | +43% (15–70) | 2.3 | 6.2 (1.6–23.8) ** |
| Objective | 12 / 19 | 63 (38–84) | +28% (−1 to 58) | 3.5 | 3.2 (0.9–11.4) |
| Both present | 12 / 18 | 67 (41–87) | +33% (5–62) | 3.0 | 4.0 (1.1–14.6) |
| Neither | 6 / 21 | 29 (11–52) | — | — | Ref. |

*Each row compares patients with the indicated finding to all patients in the comparative cohort who lack that finding. NNR = 1 ÷ absolute-risk-increase. ** Statistically significant at p < 0.05 (Fisher exact). ARD = absolute risk difference; CI = confidence interval; NNR = number needed to remove; Ref. = reference.*

**Supplemental Table S2. Sensitivity analysis of operative time (Welch t-test)**

| **Timed Cases Only** | **ML n** | **ML Mean (min)** | **LO n** | **LO Mean (min)** | **Δ (LO − ML)** | **95% CI for Δ** | **Welch p** |
| --- | --- | --- | --- | --- | --- | --- | --- |
| All cases with recorded surgical time | 19 | 122.9 | 13 | 97.3 | −25.6 | −52.7 to 1.5 | 0.063 |
| Excluding open fractures | 15 | 119.9 | 12 | 96.8 | −23.1 | −52.8 to 6.6 | 0.122 |

*Operative time is the recorded surgical case time for the index procedure; surgical case time was unavailable for 10 ML cases, which are excluded. Open-fracture status per the study database. In neither analysis did the difference reach statistical significance. ML = medial-lateral; LO = lateral-only; Δ = difference (LO − ML); 95% CIs derived from unequal-variance (Welch) t-statistics.*
